# Submerged Bioreactor Production of *Geobacillus stearothermophilus* ATCC 7953 Spores for Use as Bioindicators to Validate Hydrogen Peroxide Inactivation Processes

**DOI:** 10.3390/mps4030063

**Published:** 2021-09-10

**Authors:** Philipp Stier, Ulrich Kulozik

**Affiliations:** TUM School of Life Sciences, Technical University of Munich, 85354 Freising, Germany; ulrich.kulozik@tum.de

**Keywords:** aseptic production, product safety, sterilization, validation

## Abstract

In the food and pharmaceutical industries, evaluating the sterilization performance preceding aseptic production processes is of central importance. In the case of hydrogen peroxide sterilization of solid surfaces, bioindicators (BI) consisting of spores of *Bacillus atrophaeus* or *Geobacillus stearothermophilus* are used to validate the effectiveness and efficiency of the inactivation procedure. Commercial production of *G. stearothermophilus* is commonly performed on agar plates, where cultivation and sporulation conditions are not well-defined. Therefore, the produced BI can vary in their resistance, which in turn creates unacceptable uncertainties in the evaluation of aseptic processes. Submerged production in the bioreactor would allow more control over sporulation conditions, while reducing production time, resistance variability, and avoidance of false-positive or false-negative test results. In addition, submerged production of *G. stearothermophilus* so far was a challenge to achieve sufficiently high spore concentrations for BI production. This study reports on the development of a method for submerged production of *G. stearothermophilus* spores (pH 7.0, 57 °C, 30% pO_2_) that can achieve 1.6 × 10^7^ spores/mL with a resistance against 35% H_2_O_2_ at 25 °C of D_25°C,35% H2O2_ = 73 s. This resistance ranks within the range of commercially available BI, making the results directly transferable to industrial applications.

## 1. Introduction

In the pharmaceutical and food industries, many production processes are operated under aseptic conditions, where the sterility of all materials and equipment in contact with the product is of central importance for the safety and shelf-life of the products. To ensure aseptic conditions, sterilization performance must be validated regularly under defined conditions. Bioindicators (BI), which consist of specific microorganisms highly resistant to the sterilization process, are used for this purpose. Employing such BI, it is possible to assess whether the conditions necessary to achieve sterility are applied to inactivate a specific number of germs. A commonly applied inactivation target level is a reduction by 10^5^ colony forming units (CFU). For validation of sterilization processes using hydrogen peroxide as the sterilant, BI made of spores from *Bacillus atrophaeus* (ATCC 9372) and *Geobacillus stearothermophilus* (ATCC 7953) have become widely accepted as test BI, representing high resistance, thus covering the potential worst-case scenario of natural contamination situations [1,2,3]. When validating sterilization performance with BI, it is essential that the spores used have a defined resistance to the sterilization process. Otherwise, false-positive or false-negative validation results may occur. False-positive validation results would lead to the sterilization being incorrectly considered successful. This, in turn, would lead to a high proportion of non-sterile products in regular production, a reduction in product shelf-life, and, in the worst case, a health risk for the consumer. On the other hand, false-negative validation results would result in the wrong assumption that the sterilization was ineffective. While this does not impair product safety or shelf stability, this result would trigger harsher sterilization conditions and would thus induce higher environmental impact, over-processing, and increased costs.

However, as reported by BI users associated with us and according to oral information from member companies of the ‘German Association of Machinery and Processing Equipment Manufacturers’ (VDMA e.V.), these scenarios are a real threat, as the resistance of commercial BI varies irregularly and is mostly even unknown to the user. It can be assumed that the variation in BI resistance stems from the fact that manufacturers use different cultivation methods or inconsistent, variable sporulation conditions to produce the spores, as there are no uniform standards for BI production to date. For example, the medium or spore preparation used for spore production has not been standardized, even though it is known to significantly impact the resistance to heat or low-energy electron beam (LEEB) [4,5,6]. On the other hand, BI users report, based on practical experience, that even the resistance of spores from the same manufacturer can differ from batch to batch, which is a particular problem when neither supplier nor client are aware of it. The potential reasons for these variations have not yet been sufficiently investigated. It can be assumed that, in addition to a certain natural variability of spore resistance, this is caused by even slight variations of the conditions during spore production. In particular, the production of spores by the solid-state method on agar plates is suspected of causing resistance variations because the sporulation conditions can only be poorly controlled and hardly influenced. For instance, it is possible to affect the sporulation temperature, but it is not possible to directly influence the pH during sporulation since the pH of the agar plates is only set initially and changes due to cell metabolism during incubation [7].

However, the control of these influencing factors is of decisive importance because it was already shown in some studies that sporulation conditions, temperature and pH in particular, have a significant influence on the resistance of spores, e.g., against heat [7,8,9], LEEB [6], hydrogen peroxide [10], or other chemical sterilizing agents [11], which is commonly explained by the influence of the sporulation conditions on the spore structure [12,13,14,15]. These findings clearly show that even minor variations in sporulation conditions can significantly impact resistance, making control and precise management of influencing factors all the more critical. This fact has been known for some time but is still part of ongoing research.

Submerged BI production would allow better and more precise control of the sporulation conditions. With this method, not only the sporulation temperature and sporulation pH could be controlled and influenced in real-time, but also the oxygen saturation of the medium. Moreover, on agar plates, the cells within a colony can differ strongly, depending on the position at the surface or in the center and depending on the dynamically changing population development. For *Bacillus subtilis*, motile cells and matrix formers are known to form while another portion of the cells is already sporulating [16]. This means that not all cells have the same status, and sporulation is not uniform when BI are produced on agar plates until all nutrients are eventually depleted. Compared to that, in submerged spore production, the cells are constantly mixed and are evenly supplied with nutrients and oxygen. As a result, they do not form a biofilm with highly differentiated cells and thus have a more uniform status. This means that in submerged spore production, sporulation can be induced in a more targeted manner since nutrient shortage, which is usually used as a sporulation trigger, arrives more uniformly and at the same time for all cells. Therefore, the cell population in the bioreactor sporulates within a much smaller time interval between cells. For this reason, a lower resistance variability can be expected from submerged BI production conditions.

Another advantage relevant to the BI manufacturing industry is that spore production in the submerged process in the bioreactor takes significantly less time. Spores are usually ready for harvesting after 48 h of incubation in the bioreactor, whereas spores are incubated on agar plates for approximately 10 days and have to be manually scraped off and purified from the individual, usually hundreds of agar plates. For these reasons, in some instances, BI from spores of *B. atrophaeus* are already produced in the bioreactor. Thus, submerged production of *G. stearothermophilus* spores could be a way to reduce resistance variability of commercial BI and, therefore, to eliminate the uncertainties in validation described above. However, according to practical experience from commercial BI producers (verbal information), this has not yet been satisfactorily achieved for the production of *G. stearothermophilus* spores for unspecified reasons.

In the production of *B. atrophaeus* spores, we found in preliminary experiments that media suitable for solid-state production are often qualified for the submerged spore production as well. However, in the case of *G. stearothermophilus*, this was not even remotely the case in any of the media we tested (data not shown). Instead of sporulating, the cells died in the sporulation media. Thus, unlike *B. atrophaeus*, *G. stearothermophilus* seems to have distinctly different requirements for liquid media compared to solid media. Additionally, we also found in preliminary experiments that vegetative cells of *G. stearothermophilus* had to be handled differently. In contrast to the mesophilic *B. atrophaeus*, *G. stearothermophilus* is thermophilic with no significant growth at temperatures around 37 °C and below [5]. Accordingly, the handling of the cells and the methodological procedure must be adapted and cannot simply be adopted from the production of *B. atrophaeus* spores.

We hypothesized that submerged spore production of *G. stearothermophilus* contributes to a reduction in resistance variability because of the more defined and uniform cultivation and sporulation conditions, as described above. Therefore, we considered submerged production of *G. stearothermophilus* spores as the logical development in BI production.

However, the appropriate frame of conditions must first be identified to produce *G. stearothermophilus* spores with a suitably high spore concentration. In addition, the methodology must be robust and suitable for use in commercial spore production. Commercial BI of *G. stearothermophilus* often have a concentration of 10^7^ CFU/mL with a sales volume per vial of 10 mL. In our experience and according to industry reports, commercial BI are produced at a scale of 20–30 L. Thus, if our results for the production of *G. stearothermophilus* were applied to this scale, a large spore amount with suitable concentrations could be expected, which would be highly relevant for industrial application. To achieve this goal, we developed a methodological approach for submerged *G. stearothermophilus* spore production. For this purpose, we use different media for cultivation and sporulation because sporulation is an emergency mechanism of cells, and sporulation media that reproduce these conditions are unsuitable for cultivating vital cells and increasing cell mass.

For submerged production of *B. atrophaeus* spores in a bioreactor, it is common practice to wash the inoculum before transferring the vegetative cells into the sporulation medium to remove metabolites and residues of the spent cultivation medium. On the one hand, this complex step introduces a source of variation and does not meet the demands of thermophilic *G. stearothermophilus* since the cells cool down very quickly due to washing and media changes outside the incubator. On the other hand, we suspected that there are specific triggers and metabolites in the spent medium that promote sporulation of the cells and that the washing step loses these. We therefore assumed that omitting the washing step, in addition to suitable cultivation and sporulation medium, would have a significant effect on spore yield, even though it transfers nutrients or media residues to the sporulation medium.

Thompson and Thames [17], in 1967, already produced submerged spores of *G. stearothermophilus* (ATCC 7953). According to the current view, their cultivation of the cells was relatively rudimentary in milk dilution bottles without gassing or shaking. Sporulation was then performed in a hypering flask with a stainless-steel gas (air) dispersion tube in a water bath at 60 °C. Despite this relatively simple set-up, it was possible to produce *G. stearothermophilus* spores at concentrations up to (1–2) × 10^7^ spores/mL. However, liberated spores were rarely encountered and the cells clumped together during growth. Thus, these spores would not be suitable for use as BI because cell debris, the presence of endospore mother cells remnants, and of spore agglomerates may have a significant impact on BI resistance and storability.

Nevertheless, this study showed that submerged production of *G. stearothermophilus* was possible in principle. Therefore, we developed a new method to produce spores of *G. stearothermophilus* submerged in the bioreactor according to current standards while omitting the washing step of the inoculum. Our goal was to obtain as many liberated spores as possible, suitable for use as BI.

To investigate the influence of the washing step on spore yield, *G. stearothermophilus* spores were produced using both methods (with and without the washing step of the inoculum), but with otherwise identical sporulation conditions (pH 7.0, 57 °C, minimum oxygen saturation of 30%), and spore yield was compared. To determine the resulting spore resistance compared to spores of commercial BI, a resistance test was performed in liquid 35% hydrogen peroxide at 25 °C, in a similar and standardized procedure as commonly applied in industrial BI production.

## 2. Materials and Methods

### 2.1. Strain

Spores of *Geobacillus stearothermophilus* (ATCC 7953) with a concentration of 10^7^ colony forming units (CFU)/mL were suspended in aliquots of 500 µL each in 70% ethanol and stored at −80 °C. This enabled the spores to be taken without thawing the remaining spore suspension to ensure that the spores’ properties did not change over the experimental period.

### 2.2. Media

*Geobacillus* Medium 9 (GM9) was used as the pre-culturing medium, comprised of casein peptone 16 g/L (for microbiology, Gerbu Biotechnik GmbH, Heidelberg, Germany), yeast extract 10 g/L (for microbiology, Merck KGaA, Darmstadt, Germany), glycerol 13 g/L (Rotipuran^®^ ≥ 99.5%, Carl Roth GmbH & Co. KG, Karlsruhe, Germany), KCl 2.5 g/L (ACS reagent, 99.0–100.5%, Merck KGaA), NaCl 5 g/L (ACS reagent, ≥99.0%, Merck KGaA), NH_4_Cl 2 g/L (for molecular biology, ≥99.5%, Merck KGaA), MgSO_4_ 7H_2_O 0.8 g/L (ACS, ≥99%, Carl Roth GmbH & Co. KG), MnCl_2_ 4H_2_O 0.003 g/L (ACS reagent, ≥98%, Merck KGaA), and sodium pyruvate 5 g/L (for biochemistry, ≥99.0%, Merck KGaA), pH 7.0. This medium is a custom-designed medium established in pre-work for vegetative growth of *G. stearothermophilus* ATCC 7953 (data not shown).

Submerged Sporulation Medium (SSM) modified from [17] was used for spore production in the bioreactor, comprised of casein peptone 30 g/L (for microbiology, Gerbu Biotechnik GmbH), K_2_HPO_4_ 0.3545 g/L (ACS reagent, ≥98%, Merck KGaA), KH_2_PO_4_ 0.177 g/L (ACS reagent, ≥99%, Merck KGaA), and MnSO_4_ H_2_O 0.015 g/L (≥99%, p.a., ACS, Carl Roth GmbH & Co. KG).

### 2.3. Pre-Culture

An aliquot of 500 µL of the stored *G. stearothermophilus* ATCC 7953 spore suspension was inoculated in 400 mL of GM9 in a 1 L baffled shaking flask (Schott Duran^®^ baffled culture flask, Erlenmeyer shape, straight neck for metal caps; Duran Group GmbH, Mainz, Germany). Incubation took place at 57 °C, 150 rpm, for 16 h. After incubation, the optical density at 600 nm (OD_600_) was 3.9. Following the methodological approach from Thompson and Thames [17], the pre-culture was not washed but only homogenized and inoculated into the bioreactor, as described in Section 2.4. To compare this method with standard practice, in a second experiment, we washed the cells before transferring them into the sporulation medium. This washing step was carried out by centrifugation (4000× *g*, 10 min, 25 °C) of the pre-culture, followed by discarding the supernatant and resuspending the vegetative cells with Milli-Q water (25 °C).

### 2.4. Submerged Spore Production

One goal was to make sporulation conditions in submerged production as controllable as possible. This was achieved by automatically controlling and influencing the sporulation pH, sporulation temperature, and oxygen saturation in the sporulation medium. Second, our methodological approach should reflect the state-of-the-art and industrial BI production. This is particularly important for ensuring that the experimental results can be transferred to industrial applications without any hurdles. Additionally, it will allow us to compare the resistance of the obtained spores with commercial BI.

For this reason, we used a 2 L laboratory bioreactor (Sartorius Stedim Biostat^®^ A, Sartorius AG, Göttingen, Germany) for spore production. The bioreactor was equipped with two 6-blade disk stirrers, a flow breaker, a sparger, a heating jacket, a cooling finger, an anti-foam probe, a pH probe, a pO_2_ probe (measuring oxygen saturation), exhaust air cooling, and real-time control of oxygen saturation, pH, and temperature. The stirring speed and the gassing rate (gassing with air) were controlled automatically depending on the preset minimum oxygen saturation in the medium. If the oxygen saturation in the medium fell below the minimum value, the gassing rate increased. An increase in the stirrer speed was not necessary for the experiments carried out. The anti-foam agent Korasilon FG 30 (concentration of 3 mL/500 mL Milli-Q water, Kurt Obermeier GmbH & Co. KG, Bad-Berleburg, Germany) was added, fully automatically, as needed. The pH was adjusted fully automatically with 0.5 M HCl (ACS reagent, 37%, Merck KGaA) and 0.5 M NaOH (≥98%, pellets (anhydrous), Merck KGaA).

For spore production without the inoculum washing step, the pre-culture was inoculated directly at a ratio of 1:3 into the SSM provided in the sterilized bioreactor (total volume 1 L), and incubation was started. The OD_600_ at the beginning of the spore production in the bioreactor was 1. For the spore production method that includes the inoculum washing step, the washed pre-culture was also inoculated to an OD_600_ of 1 (total volume 1 L). The minimum oxygen saturation was set at 30%, pH at 7.0, and temperature at 57 °C. The incubation time was 48 h, sufficient for sporulation and maturation of spores. These optimal sporulation conditions were determined in preliminary experiments. Subsequently, the spores were harvested and purified. The spore production was performed as a biological duplicate.

### 2.5. Spore Harvesting and Purification

**Figure 1:**
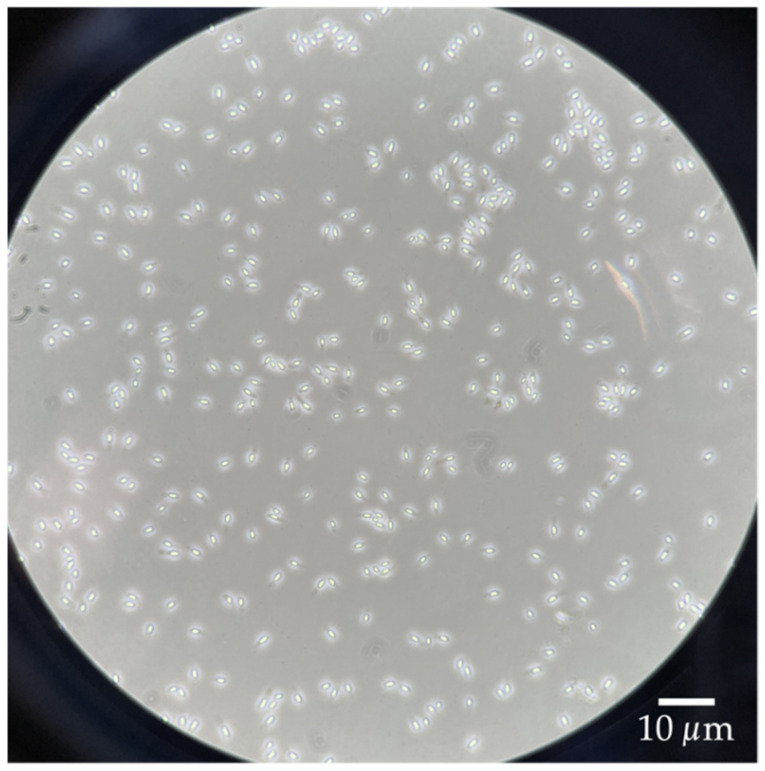
Purified spores of *G. stearothermophilus* (ATCC 7953) under the phase-contrast microscope (100×).

After incubation and sporulation of the cells in the bioreactor, the suspensions were entirely harvested. The obtained spore suspensions were further purified, largely according to [18], by separating the cell debris, endospore-containing mother cells, and liberated spores by several washing, mixing, and centrifugation steps. For this purpose, the suspension of each spore production run was divided equally into two 1 L centrifuge bottles and centrifuged at 4000× *g* for 10 min at 4 °C. After discarding the supernatants, the pellets were resuspended with 70 mL of 4 °C cold Milli-Q water each and equally divided into four 50 mL centrifuge tubes, resulting in 35 mL suspension in each centrifuge tube. After another centrifugation at 4000× *g* for 10 min at 4 °C, the supernatants were again discarded, and the pellets were resuspended with 35 mL each of fresh, 4 °C cold Milli-Q water. This washing step was repeated three to five times until the supernatant was clear. Then, after another centrifugation at 4000× *g* for 10 min at 4 °C, the pellets of the four centrifuge tubes were transferred in equal amounts into two 50 mL centrifuge tubes by discarding the supernatant and resuspending and combining the pellets with 5–10 mL of Milli-Q water. The centrifuge tubes were then made up to 40 mL with 4 °C cold Milli-Q water. The suspensions were shaken overnight at 480 rpm at 4 °C to help break down any remaining agglomerates consisting of cell debris and spores. The next day, the suspensions were centrifuged one more time (4000× *g* for 10 min at 4 °C), and the supernatant was discarded and resuspended with 35 mL of 4 °C cold Milli-Q water. In a final centrifugation step, the suspensions were centrifuged at 6000× *g* for 90 min at 4 °C, resulting in the formation of solid pellets. These pellets consisted of three phases: the top phase containing cell debris, the middle phase containing endospore-containing mother cells, and the bottom phase having pure spores. Since the sedimented pellet was very firm and the upper two phases less firm with a rather slimy consistency, the phases could be well-separated after centrifugation. For this purpose, first, the supernatant was discarded, and carefully, 3–5 mL of 4 °C cold Milli-Q water was added to the pellet and mixed vigorously. The vigorous mixing caused the two upper phases to separate slowly from the lower phase. Unlike the purification of *B. atrophaeus* by the same method, the upper phase of *G. stearothermophilus* was slightly more solid. If, in this case, the vigorous mixing was not sufficient, the phases were scraped off very carefully with a pipette tip and then washed further with some fresh, 4 °C cold Milli-Q water and vigorous mixing. The pure spore pellet was then resuspended with 45 mL of 4 °C cold Milli-Q water. The successful purification was confirmed using phase-contrast microscopy to verify that the spore suspension contained only liberated spores (Figure 1). The purified spore suspension was stored at 4 °C under the exclusion of light for further tests.

### 2.6. Determination of the Spore Resistance

To measure the resistance of spores, we determined the D-value, which describes the time required to inactivate 1-log of the spore population under constant inactivation conditions. We determined the D_25°C,35%H2O2_-value in liquid 35% hydrogen peroxide (OXTERIL^®^ 350 Spray, Food Grade, Evonik Industries AG, Essen, Germany) at 25 °C, in analogy to [18]. For this purpose, after approximately one week of storage, the purified spore suspension was first homogenized by vigorous mixing for 1 min, treatment in an ultrasonic bath for 20 min, and vigorous mixing for another minute. Then, 100 µL of this suspension was transferred into 9.9 mL of liquid 35% hydrogen peroxide, which was previously adjusted to a constant temperature of 25 ± 0.1 °C in a thermomixer. Inactivation in the hydrogen peroxide took place at 450 rpm to ensure uniform distribution of spores in the sterilant. At defined time intervals, after 30, 60, 90, 120, 180, and 300 s, 100 µL of the sample was drawn and transferred to a test tube filled with 9 mL of Ringer’s solution, 800 µL of Milli-Q water, and 100 µL of 1:5 diluted catalase (Catalase from *Micrococcus lysodeiktikus*, solution, activity 65,000–150,000 U/mL, Merck KGaA). The substantial dilution of the sample and the presence of catalase, which enzymatically degrades the hydrogen peroxide, instantaneously stopped the inactivation reaction. The catalase concentration was chosen to stop the inactivation reaction as quickly as possible without causing the test tube to foam over due to the release of oxygen from the degradation reaction. Dilutions were prepared from the samples in Ringer’s solution and plated out on Plate Count Agar (casein peptone 0.5%, for microbiology, Gerbu Biotechnik GmbH; yeast extract 0.25%, for microbiology, Merck KGaA; glucose 0.1%, for biochemistry, Reag. Ph Eur., 97.5–102.0%, Merck KGaA; agar-agar 1.5%, for microbiology, Carl Roth GmbH & Co. KG; pH 7.0) with a Drigalski spatula to determine the number of spores still capable of germination. To determine the spore concentration at time 0 s, i.e., before inactivation, the untreated suspension was also diluted and plated out. These cells were treated in the same way as the other samples, except for treatment with hydrogen peroxide. Incubation was performed at 57 °C for a total of 48 h, and colonies were counted after 24 and 48 h. The total cell count after 48 h was used for the determination of the inactivation curve. The agar plates were packed in plastic bags during incubation to prevent the agar plates from drying out due to the relatively high incubation temperature. The optimal incubation conditions of 57 °C and pH 7.0 were intended to ensure no inhibitory effects due to suboptimal recovery conditions, described in [19]. The resistance test was performed in a technical triplicate. From the obtained colony counts of the dilutions, the mean value was formed and taken logarithmically as a function of the inactivation curve. The D_25°C,35%H2O2_-value corresponds to the negative reciprocal slope of a best-fit straight line through the measurement data points.

## 3. Results

The spore suspensions from the two different methodological approaches, with and without washing the inoculum before transfer in the sporulation medium, already differed by an extreme color difference. The spore suspension in which the inoculum was washed before inoculation into the sporulation medium, according to the classical approach, was significantly lighter in color than the spore suspension inoculated without the washing step. In the purification process, the normally three-phased pellet, obtained before the final step of purification, showed only two phases. The bottom phase, which usually contains free spores, was utterly absent in the classical approach. Examination under the phase-contrast microscope confirmed that the suspension had only cell debris and endospore-containing mother cells. In contrast, the suspension in which the inoculum was not washed showed three phases in the pellet, as expected. Examination under a phase-contrast microscope showed that the bottom phase consisted of liberated spores. In addition, the pellet was almost twice as large as the pellet of the classical approach, which explains the darker color of the suspension due to the higher spore concentration overall (endospores and free spores).

Since there were no liberated spores in the pellet of the classical methodological approach, the number of germinable spores could only be determined with the endospore-containing mother cells, and was (1.0–1.1) × 10^6^ CFU/mL on Plate Count Agar. To compare this value with the suspension obtained by omitting the washing step of the inoculum, the number of germs from the two bottom phases, i.e., endospore-containing mother cells and liberated spores, was determined for this suspension, as well. The number of germinable spores was about four times higher, at (3.9–4.1) × 10^6^ CFU/mL. The determination of the spore concentration of liberated spores, calculated back to the unpurified suspension, resulted in a yield of liberated spores of approximately 1.6 × 10^7^ CFU/mL in the bioreactor. This result clearly shows the superiority of the developed production method in terms of spore yield, in which the inoculum was not washed before transfer into the sporulation medium.

Sporulation of cells represents an emergency mechanism for surviving adverse environmental conditions. Thus, one might think that the cells should have a higher number of spores, separated from the remaining nutrients of the cultivation medium due to the washing step and inoculated into a medium optimized for sporulation. Moreover, the washing step inevitably cools the suspension, representing an additional deterioration of the environmental conditions.

However, it cannot be conclusively stated why it is that the method leads to the formation of larger quantities of spores without this washing step of the inoculum. It can be assumed that, as mentioned above, metabolites and triggers are contained in the cultivation medium, which promote sporulation and are thus carried over into the sporulation medium. Possibly, the cooling of the suspension by the washing step also leads to the fact that the cells are weakened and lyse instead of sporulating in the sporulation medium, which is not suitable for the pure cultivation of vegetative and vital cells by its nature. Thus, it appears that the otherwise uncommon transfer of spent cultivation medium results in significantly improved sporulation. Nevertheless, it must be mentioned that this changes the composition of the sporulation medium in an undefined way. In the case of BI production, however, this circumstance is of minor importance if the same methodical sequence is always followed from batch to batch, and is an acceptable disadvantage, in view of the possibility of submerged production of liberated spores.

**Figure 2:**
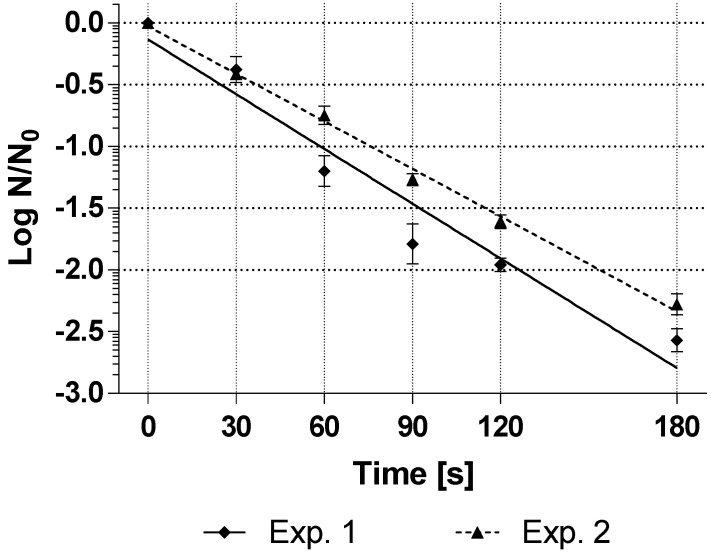
Inactivation curves with treatment with 35% hydrogen peroxide at 25 °C (technical triplicate) of *G. stearothermophilus* (ATCC 7953) spores produced submerged in the bioreactor at a sporulation temperature of 57 °C, sporulation pH of 7.0, and 30% minimal oxygen saturation. These spore suspensions were prepared without washing the inoculum before transferring into the sporulation medium. Some error bars are smaller than the symbols and thus not visible.

The next step was to investigate whether the spores obtained had a resistance to hydrogen peroxide that was suitable for use as BI in the validation of sterilization processes. For this purpose, the resistance of the self-produced spores was compared with the resistance of suspended commercial spores. Since only liberated spores can be used to determine resistance, as the cell debris and residues of the mother cells could influence the resistance test, only the resistance of the spores from the modified production method could be subjected to a resistance test. For this purpose, the purified liberated spores (total amount of 90 mL per batch with a concentration of (1.7–1.8) × 10^8^ CFU/mL) were exposed to liquid 35% hydrogen peroxide at 25 °C, and the resistance was determined as D_25°C,35%H2O2_-value from the inactivation curve. Figure 2 shows the inactivation curve of the two spore production runs in which the inoculum was not washed before inoculation into the sporulation medium.

The treatment with hydrogen peroxide caused a linear inactivation of the spore suspensions of *G. stearothermophilus* (ATCC 7953). The average resistance of the technical triplicates of the spore suspensions was D_25°C,35%H2O2_-value = 68 s for experiment 1 and 78 s for experiment 2 of the double determination, which can be considered very close to each other.

**Figure 3:**
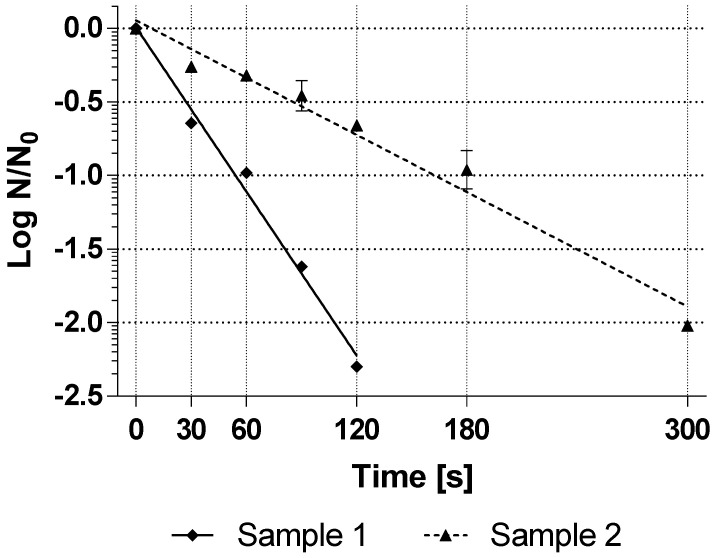
Inactivation curves with treatment with 35% hydrogen peroxide at 25 °C (technical triplicate) of BI samples of *G. stearothermophilus* (ATCC 7953) spores in suspension from two different batches of the same BI manufacturer. Some error bars are smaller than the symbols and thus not visible.

For comparison with the resistance of commercial spores, the resistance of two BI spore suspensions of *G. stearothermophilus* (ATCC 7953) from an established BI manufacturer, without a declaration of a D_25°C,35%H2O2_-value, was determined in the same way. Figure 3 shows the inactivation curves of both BI as an average of the technical triplicates.

The average resistance of the commercial BI was D_25°C,35%H2O2_-value = 54 s for sample 1 and 154 s for sample 2. These resistance values are very far apart, assuming that the BI manufacturer probably did not change the method of spore production between these two batches. As described at the beginning, this showed that the resistance of commercial spores can vary greatly not only from manufacturer to manufacturer but also from batch to batch.

## 4. Discussion

Considering that the commercial BI shown in Figure 3 are regularly used for the validation of sterilization processes, this result also showed that the spores produced submerged in this study according to the developed method are in the resistance range against hydrogen peroxide that the industry expects for BI, but with lower variability within the batches. However, it is not known under which sporulation conditions and sporulation medium the commercial spores were produced. Therefore, no deeper comparison is possible.

In a recently published study [20], we produced spores of *G. stearothermophilus* (ATCC 7953) on agar plates. For spores produced under equal sporulation conditions (57 °C, initial pH of the sporulation agar of 7.0), but with the solid-state method on agar plates, we obtained a D_25°C,35%H2O2_-value of 293 s on average, determined with the same resistance test described in this study. The resistance showed a difference in the D_25°C,35%H2O2_-value of up to 39 s within the experiments, although meticulous care was taken to avoid unwanted influencing factors during preparation causing resistance variability. For example, even the smallest differences in the pH of the medium were avoided, and the methodological sequence, media preparation, sporulation conditions, the purification process, and storage conditions where strictly adhered to. However, the resistance was significantly higher than that of the spores produced submerged. This can be justified by the fact that the spores were produced with a different medium as sporulation agar and, of course, with another production method for the reasons mentioned above. However, this shows that the variability even under experimental conditions is significantly greater for solid-state produced spores than for submerged produced spores, where the difference in D_25°C,35%H2O2_-value was only 10 s. This confirms the superiority of submerged spore production over solid-state production in terms of reducing resistance variability.

## 5. Conclusions

By the newly developed method for submerged production of *G. stearothermophilus* spores in the study presented, suitable spore concentrations and resistances could be obtained for use as BI against hydrogen peroxide. The key findings are that the proposed method obtained spores with concentrations of 10^7^ CFU/mL, making the method directly transferable to industrial application. The resistance of the produced spores lies precisely in the resistance range with which BI users are already working, which means that there are no uncertainties when BI producers would have to switch to other resistances. Additionally, this method made it possible to significantly reduce resistance variability compared to solid-state production on agar plates, increasing the safety of using the spores as BI and reducing the occurrence of false-positive and false-negative validation results. The results obtained are thus of great interest both for industrial application and in terms of scientific aspects. In future studies, it has to be clarified to what extent resistance variabilities can be further reduced by submerged spore production. The first cornerstone in this direction has been laid with the described method. To further reduce uncertainties in the validation of sterilization processes, the standardization of BI production, i.e., the definition of methods, sporulation conditions in spore production, or establishing a standard resistance of BI for sterilization with hydrogen peroxide, is necessary.

## Data Availability

The data presented in this study are available upon request from the corresponding author.
